# Extraction, purification, biological effects and applications of acrosin: a review

**DOI:** 10.3389/fcimb.2025.1596356

**Published:** 2025-06-17

**Authors:** Run Quan, Xiaolong Wu, Junjie Zhang, Rongxu Liu, Jianchun Han, Danyi Liu

**Affiliations:** ^1^ College of Food Science, Northeast Agricultural University, Harbin, China; ^2^ Heilongjiang Institute of Green Food Science, Northeast Agricultural University, Harbin, China

**Keywords:** acrosin, extraction, purification, acrosin inhibitor, enzymatic properties, reproduction

## Abstract

Acrosin is a proteolytic enzyme in the sperm acrosome that can stimulate sperm to penetrate the zona pellucida, causing the fertilization of the oocyte. Its activity is a crucial indicator of the sperm’s fertilization ability, which is critical in mammalian and human reproduction. However, there exists a lack of a comprehensive review of acrosin. In this study, we compared the extraction methods of acrosin, including acid extraction, buffer extraction, and saline extraction. The main methods for purifying acrosin, such as ammonium sulfate precipitation, ultrafiltration, dialysis, gel filtration chromatography, ion-exchange chromatography, and affinity chromatography, are reviewed. In addition, a detailed overview of the biological functions, inhibitors and applications of acrosin are outlined. This study provides methods for the extraction and purification of acrosin and some theoretical basis for the study of its properties. This provides a reference for further research on acrosin.

## 1 Introduction

Acrosin is a trypsin-like serine protease that binds as a zymogen to the inner membrane of the sperm acrosome. It is more hydrophobic than trypsin and specifically recognizes peptide bonds formed with Arg and Lys and their substrates, such as amides or esters. Acrosin is crucial in the fertilization of animals because of its unique structural properties.

As early as 1935, Yamane (Jinshin, 1935) were the first to isolate substances that can dissolve ovarian cytoplasm in the sperm of male rabbits and observe the hydrolysis of zona pellucida by sperm extracts. (Srivastava et al., 1965a; Srivastava et al., 1965b) identified these extracts as an acrosomal protein hydrolase that had a solubilizing effect on the zona pellucida of rabbit oocytes. (Stambaugh et al., 1969; Stambaugh and Buckley, 1968). named the protein hydrolase acrosin, which was found to exhibit trypsin-like properties, and trypsin inhibitors could inhibit *in vitro* fertilization. Some researchers have summarized and compared the extraction and purification methods of mammalian acrosin, such as rabbits, boar and bovine (Adham et al., 2013; Cesari et al., 2004; Zigo et al., 2011). Then, acrosin was extracted successfully from avian animals (Sasanami et al., 2011). This established the basis for the further study of the properties and applications of acrosin. In later studies, researchers discovered that acrosin had the catalytic triad structure of trypsin. Moreover, acrosin facilitated the release of other proteases from the interior to the exterior of the acrosome to allow sperm to cross the zona pellucida to complete fertilization (Isotani et al., 2017). The biological activities of acrosin and its potential applications in mammalian reproduction are gradually being emphasized by researchers (Aguirreburualde et al., 2012; García et al., 2012).

**Figure 1:**
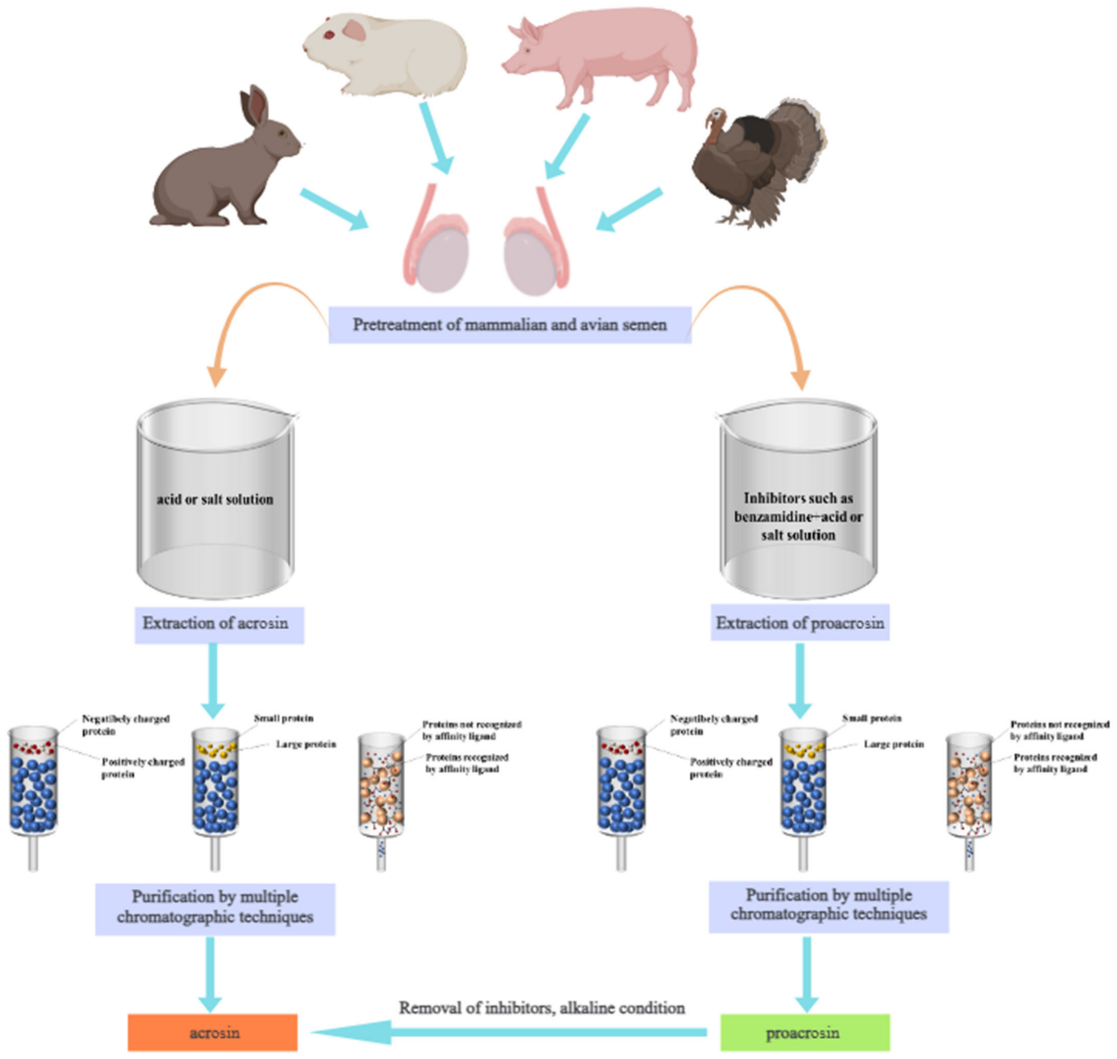
Extraction and purification process of acrosin and proacrosin.

The researchers mainly extracted acrosin by acid, buffer and saline. Then, the acrosin was purified by chromatography. This provides the basis for the subsequent study of its structure and properties. Currently, acrosin has been used in applications such as semen cryopreservation (Pan et al., 2015), mammalian fertility evaluation (Kherraf et al., 2017; Xu et al., 2018b), and the formulation of novel contraceptives (Gupta et al., 2005). In this study, we summarized the biological functions and properties of acrosin. Then, the extraction and purification methods (**Figure 1**) and applications of acrosin were reviewed. It provides a reference for further research on acrosin.

## 2 Classification of acrosin

### 2.1 Mammalian acrosin

Since the discovery of acrosin, mammalian acrosin has received extensive attention and study, especially for their role in fertilization. Mammalian acrosin has been identified and purified from rabbits (Huang-Yang and Meizel, 1975), boars (Schiessler et al., 1972), bovines (Nagdas, 1992a), goats (Brown et al., 1975), and hamsters (Siegel et al., 1986). These are primarily alkaline proteases whose activities are inhibited by acid and strong alkali (Muller-Esterl and Fritz, 1981).

Mammalian acrosin has a function in promoting hydrolysis of zona pellucida to accomplish sperm fertilization. However, this role is species dependent (Urch et al., 1985). found limited hydrolysis of zona pellucida by boar acrosin (Baba et al., 1994). found that acrosin is not required for fertilization in mice. However (Adham et al., 1997), provided the evidence of delayed fertilization in acrosin gene-deficient mice by disrupting the mice acrosin gene. In a recent study, acrosin was found to be essential for fertilization in hamsters (Hirose et al., 2020). These studies suggest that acrosin is not broadly essential for fertilization in mammals, but that the function of acrosin depends on the species (Yamagata et al., 1998). showed that the lack of the acrosin gene caused a decrease in the diffusion rate of mouse acrosomal proteins, which finally delayed the penetration of mouse sperm into the zona pellucida. In a recent study, limited hydrolysis of the zona pellucida by mouse acrosin affected its mechanical resilience and binding to sperm (Kuske et al., 2021). Acrosin does not hydrolyze the zona pellucida through simple degradation but partial hydrolysis to change its structure and promote sperm fertilization.

Mammalian acrosomal enzymes also share some structural similarities. Caprine acrosin can solubilize the zona pellucida of sheep, pig, and mouse oocytes (Brown, 1982). The mammalian acrosin from rabbit, bovine, boar, sheep, and stallion was detected through immunofluorescence using bovine acrosin antibody as an antigen marker, indicating that a certain similarity exists in the structure of acrosin from these mammals. In addition, some researchers have found that proacrosin from boars, bovines, hamsters, rams, and humans could bind to the polyclonal antibody of boar tproacrosin-binding protein, further demonstrating the similarity in the structure of mammalian acrosin (Yi and Polakoski, 1992).

### 2.2 Avian acrosin

Current studies on avian acrosin have focused on some poultry acrosin, such as chicken, turkey, and quail (Langford and Howarth, 1974). isolated a trypsin-like enzyme from chicken, turkey, and quail sperm (Froman, 1990). demonstrated that this protease was an acrosin. Subsequently (Richardson et al., 1988), discovered that turkey acrosin, like mammalian acrosin, is a glycoprotein with serine protease characteristics. Hence, poultry acrosin is possibly a trypsin-like serine protease. The structure of turkey acrosin has been extensively studied. It comprises light and heavy chains forming a double-stranded molecule whose cDNA fragment comprises signal and proacrosin residue sequences (Slowinska et al., 2010). Japanese quail sperm acrosin stimulates sperm binding to the vitelline membrane and accelerates the hydrolysis of its glycoproteins, similar to mammalian promotion of sperm penetration through the zona pellucida (Sasanami et al., 2012, 2011).

### 2.3 Aquatic animals acrosin-like enzymes

Compared to those of mammalian and avian acrosomal proteins, less research has been done on acrosin-like enzymes in aquatic animals. Some studies have identified the presence of proteases in aquatic animals sperm that facilitate sperm penetration of the zona pellucida (Levine and Walsh, 1980). purified these proteases from sea urchin sperm and found that their activities can affect fertilization in sea urchins. This protease has two subunits (34 kDa and 18 kDa). One of the 34 kDa subunit has the catalytic activity of an acrosomal enzyme. However, the 18 kDa subunit seems to have activity to inhibit hydrolysis. Hence, they were named acrosin-like enzymes.

The acrosin-like enzyme of lake sturgeon sperm has similar enzyme activities that are restrained by mammalian acrosin inhibitors. This suggests that acrosin-like enzymes of aquatic animals have many similarities with mammalian acrosin (Ciereszko et al., 1996). However, two trypsin-like proteases have been identified in ascidian sperm, one of which has been found to be highly similar to mammalian acrosin through cDNA cloning. The other is a novel trypsin-like enzyme, and both proteases are involved in fertilization (Kodama et al., 2002, 2001). The acrosin-like enzymes of aquatic animals and mammalian acrosin share many features; however, some unique properties possibly result from structural change adaptation in the fertilization environment.

## 3 Properties of acrosin

### 3.1 Structural properties

**Figure 2:**
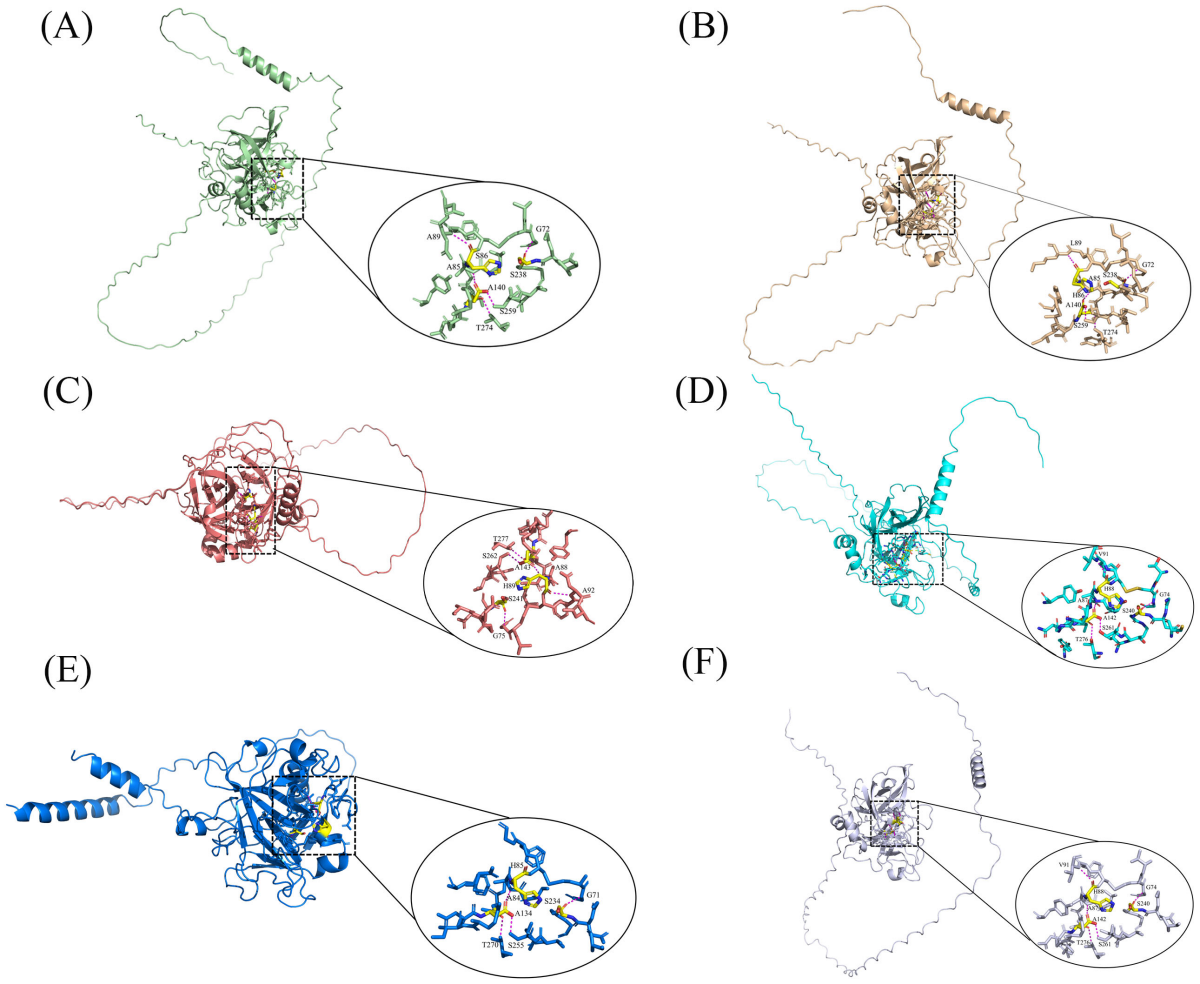
3D structure and active site of acrosomal enzymes from different animals Detailed view of the active sites with acrosin is displayed on the ellipse. The yellow area indicates the active site, and the magenta dashed line indicates the force of interaction between the active site and the ligand. **(A)** Rabbit; **(B)** Pig; **(C)** Rat; **(D)** Human; **(E)** Wild turkey; **(F)** Quail (Bateman et al., 2024).

Acrosin is the most studied and characterized acrosomal protease (Moreno and Alvarado, 2006). Currently, the acrosin genes of mice, rats, humans, and pigs have been characterized and are located on Chromosome 15, 7, 22, and 5, respectively (Adham et al., 1991; Kremling et al., 1991; Yasue et al., 1999). Researchers have currently deduced the amino acid sequences of human (Baba et al., 1989c), boar (Huh and Yi, 2001), bovine (Adeniran et al., 1995), mouse (Klemm et al., 2010), and turkey (Slowinska et al., 2010) acrosin using cDNA cloning and genetic sequence. Acrosin exists as an inactive proacrosin on the acrosomal inner membrane before the acrosome reaction occurs. Proacrosin comprises a signal peptide, pro-peptide, catalytic domain, and C terminal domain. After the acrosome reaction occurs, proacrosin is hydrolyzed by its proteolytic enzymes through an intra-enzymatic mechanism into a stable and enzymatically active acrosin (Slowinska et al., 2010), which is partially conserved in its structure in birds and mammals (Berlin et al., 2008). It has been demonstrated that the structure of acrosin comprises the zymogen domain, catalytic domain, and tail structure using genome evolution and cDNA cloning (Fock-Nuzel et al., 1980). Its zymogen and catalytic domains, particularly the catalytic triad site in the catalytic domain, that is, His, Asp, and Ser, have high conservations of acrosin and serine proteases of the S1 family in different species (**Figure 2**) (Klemm et al., 1991). However, the tail domain of acrosin from various species have different structures and is highly variable. The molecular weight of acrosin is the same as that of some serine proteases, and the sequence of some amino acid residues at the N-terminal end is very similar to that of some serine proteases, such as plasmin and chymotrypsin (Raterman and Springer, 2008; Smith et al., 2000). Acrosin is an alkaline protease, and boar acrosin has an isoelectric point of 10.5, with stabilization in neutral and slightly alkaline environments and high enzyme activity, whereas acidic environments inhibit enzyme activity (Hardy et al., 1991; Muller-Esterl and Fritz, 1981; Westbrook-Case et al., 1994).

Acrosin comprises two chains, the heavy and light chains, which are connected by two disulfide bonds (Jones et al., 1988; Polakoski and Parrish, 1977). First, cleavage of Arg-23 and Val-24 of proacrosin causes the formation of a double-stranded polypeptide intermediate with amino acid terminal sequences identical to the light and heavy chain sequences of the acrosin, that is, the formation of the light and heavy chains. Subsequently, the 85 amino acids at the C-terminal are hydrolyzed. Finally, the activation of the zymogen, which has the same amino acid terminal sequence as the acrosin, to the acrosin occurs (Baba et al., 1989a; Zahn et al., 2002). In the activation process of porcine sperm, proacrosin, which has a molecular weight of 55 kDa, is converted to acrosomal enzymes with molecular weights of 34 and 32 kDa (Sun et al., 2013).

### 3.2 Enzymatic properties of acrosin

Acrosin has two action sites; the first is the trypsin catalytic site, giving it the enzymatic activity of trypsin; the other is a positively charged region close to the catalytic site, which may be related to the binding of zona pellucida glycoproteins. It has also been found that acrosin demonstrates a lectin-like carbohydrate-binding activity, which causes sperm that had undergone an acrosome reaction to bind to the zona pellucida in a non-enzymatic binding (Adham et al., 2013; Honda et al., 2002). Acrosin has a catalytic triad structure with His, Asp, and Ser sites, which can selectively hydrolyze the Arg-Xaa and Lys-Xaa bonds. It strongly hydrolyses the amide and ester substrates of Arg and Lys; however, its hydrolytic activity for amide and ester substrates of Asp is stronger (Cesari et al., 2010; Töpfer-Petersen et al., 2008).

When the sperm cell undergoes the acrosome reaction, proacrosin is activated into the acrosin with enzymatic activity. However, this activation process is inhibited by benzamidine, p-aminobenzamidine, 4-(2-aminoethyl) fluoride hydrochloride, soybean protease inhibitors, etc., and the acrosome reaction induced by the capacitated sperm is delayed by these trypsin inhibitors. Since proacrosin cannot be converted to acrosin in a timely manner, the dispersion of the acrosomal matrix outside of the acrosome is delayed, causing the failure of oocytes to fertilize properly (Beek et al., 2015b; Straus and Polakoski, 1982; Tollner et al., 2000). Some metal ions, such as Ca^2+^ and Na^+^, can also modulate proacrosin activation (Parrish and Polakoski, 1981), while divalent metal ions, such as Zn^2+^, Cu^2+^, Hg^2+^, Co^2+^ and pb^2+^, effectively inhibit acrosin activity (Li et al., 2017; Steven et al., 1982). Temperature and pH had a relatively significant effect on the activity of acrosin, with the lowest activity of boar acrosin at pH 5.0, and the activity of acrosin increased with increasing pH, with the highest activity of the enzyme that hydrolyzes BAEE at pH 8.7; the optimum temperature of acrosin is 37°C, and the enzyme activity is inhibited at temperatures higher than 37°C. A temperature that is too high destroys the protein structure of acrosomal enzymes, and the enzyme activity is reduced by half at 55°C (Polakoski et al., 1973). In addition, mitochondrial functionality and Clock genes can regulate acrosin activity. Mitochondrial functionality can be used to modify human sperm acrosin activity, acrosome reaction capability, and chromatin integrity, and sperm samples with high mitochondrial membrane potential had significantly high mitochondrial membrane potential sperm samples had higher acrosomal enzyme activity than low mitochondrial membrane potential. The Clock gene regulates the activity of acrosin by modulating the serine esterase SEPINA3K protein (Cheng et al., 2016; Zhang et al., 2019).

## 4 Biological function of acrosin

Acrosin is located on the inner acrosomal membrane matrix as an inactive acrosomal zymogen before the acrosome reaction occurs. The acrosome reaction occurs before the sperm cell binds to the oocyte and promotes the activation of proacrosin into acrosin, which makes the enzyme active for hydrolysis (Moreno et al., 2011; Puigmulé et al., 2011). The zona pellucida glycoprotein in mammals comprises the ZP1, ZP2, and ZP3, while the human zona pellucida glycoprotein comprises four glycoproteins, namely ZP1, ZP2, ZP3, and ZPB. Acrosin conjugates to zona pellucida glycoproteins, facilitating their hydrolysis and the completion of fertilization. Several studies have found that ZP3 can induce spermatocytes to react in the acrosome reaction and promote the activation of acrosin; ZP2 binds to acrosin. Kuske et al (Kuske et al., 2021). identified the binding sites of acrosin and ZP using mass spectrometry, and the results showed that ZP1 has two acrosin binding sites, ZP2 has five, and ZP3 has one. Acrosin binds to these sites and hydrolyzes the glycoprotein, which causes the remodeling of the zona pellucida (Gupta, 2021; Howes et al., 2001; Kim et al., 2001; Kuske et al., 2021; Moros-Nicolás et al., 2021).

Only sperm with intact acrosome can cross the fallopian tube; after reaching the ampulla from the tubal isthmus, the oocyte promotes spontaneous acrosomal exocytosis of the sperm. The acrosomal membrane binds to the oocyte zona pellucida glycoprotein ZP3 to induce the sperm acrosomal reaction, in which the intra-acrosome pH rises to near-neutral conditions in the surrounding environment. Proacrosin is achieved by the removal of a C-terminal segment rich in proline residues and by the cleavage of the Arg23-Val24 bond leading to the formation of the light and heavy chains. During the acrosome reaction, proacrosin may bind to mannose residues in ZP glycoprotein through the binding sites at the N- and C-terminal ends of protein, and sperm and oocyte recognize each other (Furlong et al., 2005). The activated acrosin binds to the acrosin matrix, facilitating the release of the acrosomal matrix and other proteolytic enzyme within the acrosome to the outside of the acrosome. Hyaluronidase, dipeptidyl peptidase, etc, can be freely solubilized and rapidly released. The acrosomal components are gradually released based on their solubility (Ikawa et al., 2010; Kim and Gerton, 2003; Nagdas, 2016; Okabe, 2018). Acrosin binds to the fucose-binding protein on the outer acrosomal membrane, and the complex binds to the ZP3, which promotes acrosomal catabolism and releases proteolytic enzymes, such as hyaluronidase, some proteases of the proteasome within the acrosome, etc (Töpfer-Petersen et al., 2008). Acrosin hydrolyzed ZP2, destroying the dense structure of the zona pellucida. These hydrolytic enzymes promote the hydrolysis of zona pellucida glycoproteins, and sperm penetrate the zona pellucida of the oocyte to complete fertilization (Ferrer et al., 2012; Gahlay and Rajput, 2020; Saldívar-Hernández et al., 2015; Seol et al., 2022; Topfer-Petersen and Henschen, 1987).

Some researchers have shown that acrosin is not necessary for the process in which sperm cross the zona pellucida during fertilization to complete fertilization (Baba et al., 1994). By knocking out the acrosin gene in mice, the researchers found that mice had low fertility and could not complete *in vitro* fertilization, delaying sperm from penetrating the zona pellucida of the oocyte. However, fertilization can be accomplished in the female reproductive tract and *in vivo*, probably because the female reproductive tract compensates for some of the lack of sperm function (Hirohashi et al., 2011; Isotani et al., 2017; Kawano et al., 2010). A recent study found that sperm lacking acrosin was unable to penetrate the ZP, rather than hampering sperm binding, disrupting gamete fusion, or preventing oocyte activation. Acrosin deficiency causes total fertilization failure in humans by preventing the sperm from penetrating the zona pellucida (Hua et al., 2023). This finding suggests that acrosin may be particularly crucial for releasing hydrolytic enzymes in the acrosomal matrix and facilitating the dissolution of the zona pellucida.

## 5 Extraction of acrosin

The methods for extracting acrosin from animal testes or sperm and human semen include acid extraction, buffer extraction, and saline extraction. The extraction solvents and advantages and disadvantages of the three methods are listed in **Table 1**.

**Table 1:** Comparison of extraction methods for acrosin.

Method	Extract Solven	Specific Enzyme Activity	Advantage	Disadvantage	Refences
Acid extraction	Sulfuric acid	15.2 U/mg	Stable and easy to store	Low extraction rate	(Meizel and Mukerji, 1975)
Buffer extraction	MES buffer	12.6 mU/mg	Less impurity	Low enzyme activity	(Brown, 1983)
Saline extraction	Hyamine 2389	0.36 U/mg	Easy operation	Low enzyme activity	(Elce and Mcintyre, 1982)

### 5.1 Acid extraction

The acid extraction method is the most commonly used method for acrosin extraction. Its advantages include the ability to extract proacrosin and keep acrosin from being activated; in addition, the enzyme is stable and easy to store (NagDas, 1992a). However, its disadvantage is that the lower pH may lead to the denaturation of proteins, destroying enzyme structure and reducing enzyme viability. The acid extraction method is simpler to operate and often uses hydrochloric, acetic, and sulfuric acids. First, animal sperms are washed with NaCl or phosphate buffer to remove contaminants. Next, the decontaminated sperms are extracted in acid solution to obtain the crude enzyme solution (Polakoski and Parrish, 1977). Acid extraction has been widely used for the extraction of mammalian acrosin from rabbits, boars, hamsters, bovines, and other mammals (Cechova et al., 1988; Meizel and Mukerji, 1975; NagDas, 1992a; Siegel et al., 1986). In addition (Tobias and Schumacher, 1976), compared the extraction effect of multiple washing extraction and direct extraction with acid solution. The enzyme activity of human acrosin extracted by multiple washes was threefold higher than that of direct extraction. Multiple washes might be effective in removing plasma, acrosomal membranes, and stray proteins, leading to increased enzyme purity.

### 5.2 Buffer extraction

The buffer extraction method is used to obtain acrosin by mixing washed animal semen or animal testis homogenate with buffer solution and then filtering it out. This method uses buffers such as Tris buffer (pH 7.5) (Ferrer et al., 2012) and 2-morphine ethanesulfonic acid buffer (pH 6.0) (Brown, 1983). To reduce the heteroprotein content in the extract, the addition of Triton X-100, Triton X-114, and octyl-β-D-glucopyranoside to the buffer allows the separation of membrane proteins (Zigo et al., 2011). However, the buffer-extracted acrosin activity is lower. As shown in **Table 1**, the specific enzyme activity of buffer-extracted acrosin has been shown to be 12.6 mU/mg, which is lower than that extracted by acid (Brown, 1983). Higher pH might lead to the activation of acrosin that is damaged during extraction, thereby reducing enzyme stability.

### 5.3 Saline extraction

The saline extraction method relies mainly on the fact that when the salt concentration is low, the addition of an appropriate amount of salt solution improves the solubility of proteins; therefore, this method is usually used to extract proteins. However, too high a concentration of salt solution reduces the solubility of proteins and denatures them (Zhang et al., 2009a). The salt solutions used to extract acrosin from animal tests and semen are usually CaCl_2_, Hanks’ balanced salt solution, and Tris (Elce and Mcintyre, 1982; Sasanami et al., 2011; Yu et al., 2006). Human semen incubated at 37°C with quaternary ammonium 2389 solution containing 0.075% Triton X-100 was found to yield acrosome extract, which was centrifuged to obtain the acrosin-containing supernatant (Zaneveld et al., 1972). The salt solution extraction method can achieve a higher extraction rate of acrosin; however, the salt solution may affect acrosin structure and influence enzyme activity.

## 6 Purification of acrosin

**Figure 3:**
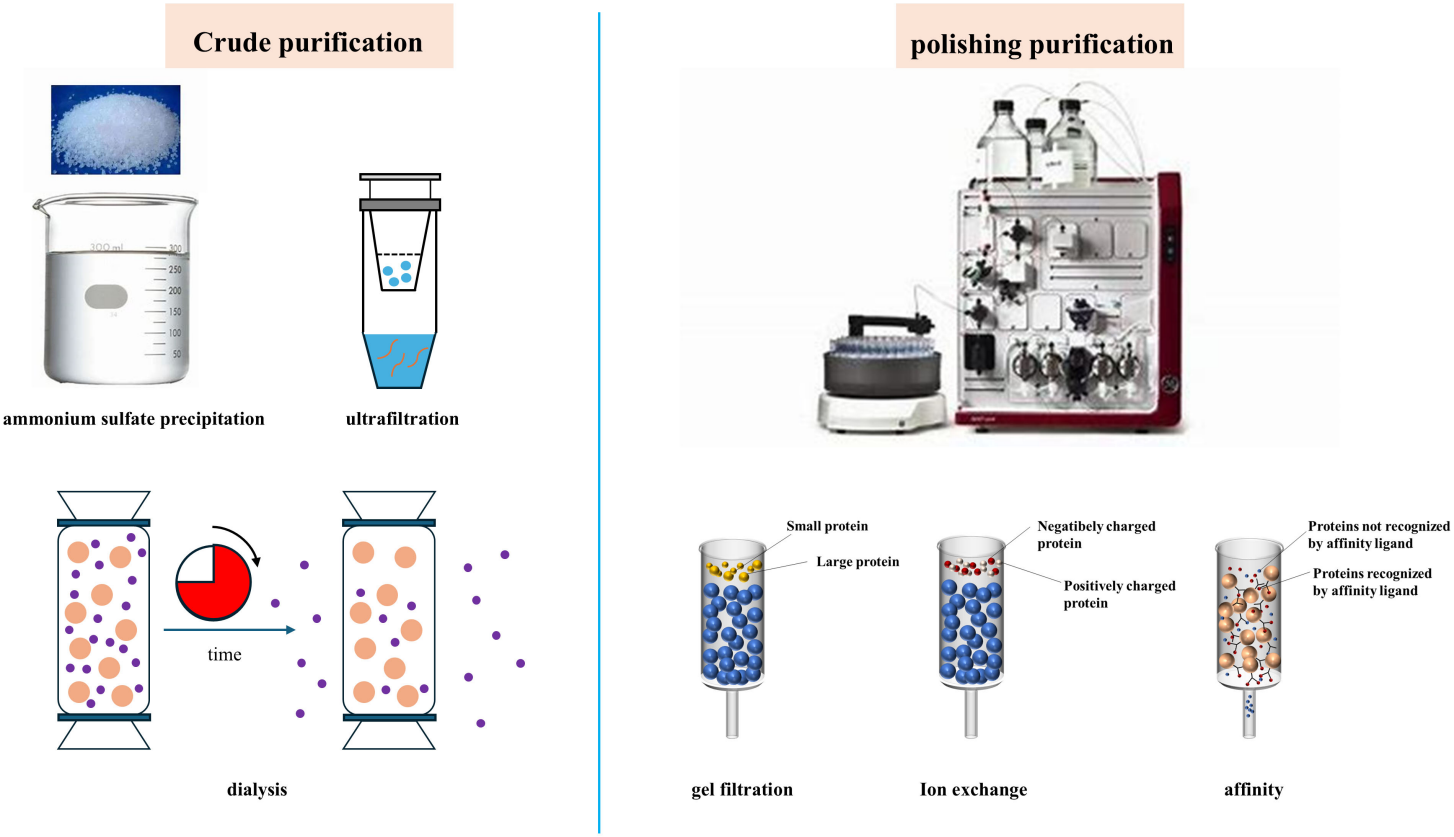
Main purification methods of acrosin.

The crude enzyme solution extracted from animal semen or testes contains not only hyaluronidase and esterase but also proteins other than acrosin, such as acrosomal proteins and membrane proteins. As shown in **Figure 3**, purification of acrosin can be divided into crude and refined purification. The crude purification of acrosin includes ammonium sulfate precipitation, dialysis, and ultrafiltration. Acrosin is typically refined purified by chromatography, including gel filtration chromatography, ion-exchange chromatography, and affinity chromatography.

### 6.1 Crude purification

#### 6.1.1 Ammonium sulfate precipitation

Ammonium sulfate precipitation is a commonly used protein separation method that removes various non-protein impurities and concentrates the target protein. The principle is that high concentrations of salt ions can compete for more water molecules, resulting in damage to the hydrated membrane structure on the protein surface, thus reducing protein solubility and causing it to precipitate out of solution (Burgess, 2009). Baba et al. (1989b) extracted crude enzyme solution from animal testes, purifying it using 50% ammonium sulfate. After chromatography, acrosin was obtained with molecular weights of 55 kDa and 53 kDa.

Graded ammonium sulfate precipitation can remove non-protein impurities and heterogeneous proteins to increase the content of target proteins. This is based on the principle that the solubility of proteins differs at different ammonium sulfate concentrations (Pringels et al., 2018). NagDas et al. (1992b) used 30% and 85% ammonium sulfate to precipitate the crude enzyme solution, and the specific activity of acrosin was found to increase by4.2-fold. Therefore, ammonium sulfate–graded precipitation is an effective crude purification method for the crude separation of acrosin.

#### 6.1.2 Ultrafiltration

Ultrafiltration is a molecular-scale membrane filtration technology that uses a pore size of 10–100 nm. The separation principle is to selectively separate solutes of different molecular weights by the pressure difference between the two sides of the membrane (Ma et al., 2024). In the context of protein separation, ultrafiltration has the advantages of mild operating conditions, protein stabilization, and high resolution (Marson et al., 2021). Therefore, it is widely used for the concentration of complex mixed samples, recovery of target products, and depyrogenation of pharmaceuticals and biochemicals (Ma et al., 2024; Zhao et al., 2023). Following acetic acid extraction of human sperm acrosin, when the crude enzyme solution was purified using ultrafiltration tubes (molecular weight cut-off value of 10 kDa) at 4°C and centrifuged at 20,000 ×g for 25 min, the total enzyme activity was found to increase by 12.2 mU (Berruti, 1980). Chromatographic purification treatments may dilute the target protein, whereas ultrafiltration treatments may concentrate the target protein and increase the protein content (Antaki et al., 1987). used ultrafiltration tubes (molecular weight cut-off value of 10 kDa) after gel filtration chromatography of the crude enzyme solution of bovine acrosin, resulting in two novel acrosins, BSP66 and BSP120.

#### 6.1.3 Dialysis

Dialysis is a method of separating proteins and small molecules. The difference in salt ion concentration exists on both sides of the semipermeable membrane, whereby small molecules spontaneously penetrate through the semipermeable membrane from the dialysate (high-concentration solution) to the low-salt solution, and macromolecules such as proteins remain within the semipermeable membrane (Gueccia et al., 2019; Zawada et al., 2022). Acrosin extraction and purification methods such as saline extraction or ammonium sulfate precipitation lead to the introduction of large amounts of salt ions, and affect enzyme properties. To prevent acrosin activation during the extraction process, acrosin inhibitors such as benzamidine are often added to the extract to maintain acrosin stability. Therefore, dialysis treatment is required to remove salt ions and chemicals. (NagDas, 1992a) washed bull sperm with NaCl-containing benzamidine and centrifuged and resuspended it in glycerol containing benzamidine with agitation for extraction. Finally, the crude enzyme solution was removed from benzamidine and salt ions by dialysis. The extraction of bovine sperm acrosin using Tris-saline-protease inhibitor solution and CAPS buffer ((3-[cyclohexylamino]-1-propanesulfonic acid) and dialysis to remove small molecules has been shown to yield acrosin and other acrosomal proteases (Nagdas et al., 2016).

### 6.2 Chromatographic purification

After the separation of acrosin by ammonium sulfate precipitation and ultrafiltration or dialysis, it is finely purified by gel filtration chromatography, ion-exchange chromatography, or affinity chromatography. **Table 2** compares the purification of acrosin by three common chromatographic techniques. Better purification can only be achieved by choosing the appropriate resin and elution buffer (Sánchez-Trasviña et al., 2021).

**Table 2:** Comparison of chromatographic techniques for acrosin.

Type of chromatography	Chromatographic Matrix	Elution Buffer	Purification Fold	References
Gel filtration	Sephadex G-100	7 M urea/1 M formic acid	Unknown	(Fock-Nuzel et al., 1980)
Gel filtration	Superdex 200	4 M urea (pH 3.0)	5.8	(Slowinska et al., 2010)
Cation exchange	SP-Sephadex	A linear concentration gradient (0.2-0.8 M) of NaCl in 0.05 M sodium acetate buffer (pH 4.2)	3.6	(Adekunle et al., 1987)
Cation exchange	CM-Cellulose 32	A linear concentration gradient (0.15-0.5 M) NaCl in 0.1 M sodium acetate buffer (pH 4.0)	23.5 (α-Acrosin), 46.5 (β-Acrosin)	(Zelezna and Cechova, 1982)
Affinity	p-aminobenzamidine CH-Sepharose 4B	50 mM triethanolamine/HCl containing 0.5 M NaCl (pH 7.9)	50	(Berruti et al., 1981)
Affinity	SBTI-Sepharose	0.5 M NaCl-50 mM TRA-HCl (pH 7.8)	135.7	(Leyton et al., 1986)

#### 6.2.1 Gel filtration chromatography

Gel filtration chromatography or particle size exclusion chromatography is a method of separating proteins based on differences in their molecular size (Mogilnaya et al., 2021). It has the advantages of high selectivity, high resolution, simple operation, and flexible changes in elution conditions and buffer components that do not affect the resolution. Therefore, it has been widely used in the separation and purification of proteins and peptides. However, this chromatographic technique has some disadvantages such as long sample collection time and high cost (Shaik and Sarbon, 2022; Wang et al., 2017). The pore size of the gel matrix has a major effect on the effectiveness of gel chromatography because it determines the size of the molecules that penetrate the interior of the gel. In one study, a crude enzyme solution of acrosin from boars was purified by Sephadex G-100 columns, and the purified product appeared as a single 38 kDa band in a polyacrylamide gel, which is a good purification result (Fock-Nuzel et al., 1980) (Slowinska et al., 2010). purified turkey acrosin by gel filtration chromatography using a Superdex 200 column and achieved 5.8-fold purification with a recovery of 24%. This purification method is useful in isolating acrosin from most sperm proteins and inhibitors.

#### 6.2.2 Ion-exchange chromatography

Ion-exchange chromatography is an effective technique for purifying animal proteases, and it is widely used in the separation of proteins and peptides and structure determination. The target protein binds to the opposite charge carried by the ion-exchange chromatography medium, and the concentration of charged molecules gradually increases when the buffer eluent or pH is changed. Proteins are separated based on their affinity for ion exchangers. Elute in a different order to purify the target protein (Taddia et al., 2021; Zou et al., 2024). Ion exchange chromatography is characterized by high selectivity and high loading and is suitable for enrichment of target products (Levison, 2003). Ion-exchange agents used for the purification of acrosomal enzymes by ion-exchange chromatography include sulfopropyl (SP) (Adekunle et al., 1987) and carboxymethyl (CM) (Leyton et al., 1986). SP is a strong cation exchanger, whereas CM is a weak cation exchanger, and the buffer pH should be at least one unit below the isoelectric point of the bound protein, with the CM ion-exchange buffer having a lower pH than that of SP (Polakoski and Parrish, 1977). purified boar acrosin by cation-exchange chromatography on CM-cellulose 32 columns. α-Acrosin (molecular weight 50 kDa) and β-acrosin (molecular weight 35 kDa) were obtained, which were purified 23.5-fold and 46.5-fold, respectively. Ion-exchange chromatography provides better purification than that of gel filtration chromatography. In addition, the technique has the disadvantages of complex operation, sensitivity to pH and metal ions, and high cost.

#### 6.2.3 Affinity chromatography

Affinity chromatography is a protein purification method based on specific and reversible binding occurring by biological interactions (Alishah et al., 2023). These interactions include those between antibodies and antigens, enzymes and substrates, and hormones and receptors, where biocorrelators that are immobilized on carriers are known as affinity ligands (Rodriguez et al., 2020). They maintain high resolution and high selectivity even in complex mixtures (Hage et al., 2012). Affinity ligands can consist of a variety of biologically relevant agents, such as antibodies, enzymes, transporter proteins, and metal ion chelates, which are critical in determining the success or failure of affinity chromatography (Hage and Matsuda, 2015). The affinity ligands commonly used for the purification of acrosomal enzymes by affinity chromatography include concanavalin A, benzamidine, and p-aminobenzamidine. Among these, concanavalin A is a lectin that reversibly binds to targets in the sugar-containing fraction (Hirabayashi et al., 2015); p-aminobenzamidine and benzamidine are competitive inhibitors of acrosin. They inhibit acrosin activity by binding non-covalently and specifically to the active site of acrosin. This binding is reversible. The property is the main reason for their use as ligands for affinity chromatography. Berruti et al. (1981) purified bovine acrosin 50-fold by affinity chromatography with p-aminobenzamidine-CH-Sepharose 4B. Mukerji et al. (1979) purified rabbit acrosin through gel filtration chromatography and affinity chromatography of concanavalin A- Sepharose columns. Two gel filtration chromatograms purified it 34.4-fold, followed by a final affinity chromatography purification of 145.6-fold. The purification performance of affinity chromatography is superior to that of the other two chromatographic techniques, and the combination of affinity chromatography and other chromatographic techniques improves purification. Moreover, soybean trypsin inhibitors and monoclonal antibodies to acrosin have been used as affinity ligands to purify acrosin with good purification and high yield (Leyton et al., 1986). Affinity chromatography has the advantages of simplicity and speed compared to other chromatographic techniques. It is very useful in the purification of numerous biomolecules, biopharmaceuticals, and other substances (Lacki and Riske, 2020).

## 7 Acrosin inhibitors

The mammalian reproductive process is co-regulated with various proteases and protease inhibitors. Studies have been used to identify 19 Adamalysin-related proteinases that are expressed in human and animal testes and participate significantly in sperm-egg recognition (Le Magueresse-Battistoni, 2008; Porter et al., 2005). The activity of these proteases is regulated by protease inhibitors. Failure of the regulatory mechanism may cause failure in the beginning and normal progression of mammalian reproduction, which may lead to infertility.

### 7.1 Synthetic acrosin inhibitors

Acrosin inhibitors can be classified into synthetic and natural acrosin inhibitors according to their source. The synthetic acrosin inhibitors include nitrophenyl p-guanidinium benzoate, benzamidine, p-aminobenzamidine, and some novel acrosin inhibitors (Llanos et al., 1993). Benzamidine and p-aminobenzamidine reduce enzyme activity by binding to the active site of the acrosin and inhibiting its binding to the substrate (Stasiak et al., 2012). p-nitrophenyl-p’-guanidine benzoate (NPGB) can block the spermatocyte acrosome reaction by inhibiting the progesterone-induced increase in the inward flow of calcium ions, resulting in a failure of the acrosome reaction to proceed normally and inhibiting acrosin activation (Slowinska et al., 2012). In contrast, others novel inhibitors directly inhibit acrosin and its zymogen activity (Zhang et al., 2009b).

Gossypol can inhibit the activity of acrosin and proacrosin, which causes the failure of the oocyte to fertilize properly, and it is a good contraceptive (Tso and Lee, 1982). Tosyl-L-lysyl Chloromethyl Ketone could inhibit the acrosome reaction of the spermatid. Similarly, it reduces the mitochondrial membrane potential and sperm motility. It affects the integrity of the membrane and acrosome and inhibits fertilization of the spermatid and oocyte. Furthermore, 4-(2-aminoethyl)-benzenesulfonylfluoride (AEBSF) can reduce sperm penetration during fertilization, which may be owing to its inhibitory effect on acrosin activity; however, it did not affect sperm motility (Beek et al., 2015a; Deppe et al., 2008).

### 7.2 Natural acrosin inhibitors

Natural acrosin inhibitors include serine protease inhibitors in mammals such as Kazal-type, Kunitz, and plasminogen activator inhibitors, antithrombin III, and SBTI. The acrosin inhibitors of mammals are primarily secreted from the seminal vesicle and are in a structural relationship with Kazal inhibitors (Watthammawut et al., 2015). The serine protease inhibitor Kazal family (SPINKs) is the largest branch of the Kazal inhibitors. Currently, SPINK1-14, SPINKL with SPINK family have been identified in different mammals (Jalkanen et al., 2006; Liao et al., 2022; Lin et al., 2008; Lu et al., 2012; Ou et al., 2010; Rimphanitchayakit and Tassanakajon, 2010). SPINK inhibits oocyte fertilization by regulating the serine protease activity. SPINK bind to sperm, reduce the inward flow of calcium ions within the sperm head, and inhibit the acrosome reaction caused by calcium ion inward flow, resulting in the inability to activate proacrosin (Ikawa et al., 2010; Okabe, 2018). The soybean trypsin inhibitor is a serine protease inhibitor in plants that can inhibit acrosin activity. Similar to AEBSF, it does not induce premature acrosome reaction and does not affect the sperm membrane integrity, mitochondrial membrane potential, and the motility parameters of the spermatid (Ou et al., 2012).

## 8 Application of acrosin

Currently, researchers have used the property of acrosin to stimulate sperm to penetrate the zona pellucida and complete fertilization for applications in sperm cryopreservation, new contraceptive development, sperm quality measurement, and male infertility diagnosis.

### 8.1 Testing of frozen sperm quality

The history of sperm cryopreservation dates back 200 years, and the discovery of freeze resistance for glycerol was a turning point for this technology. Sperm cryopreservation has currently become an essential tool for the long-term preservation of genetically superior males and transgenic and endangered species (Peris-Frau et al., 2020; Polge et al., 1949; Royere et al., 1996). Cryopreservation can damage the sperm significantly, causing cryo-damage of sperm cells and altering the activity of sperm proteins, lipids, and some enzymes. Vilagran et al (Vilagran et al., 2013). compared the activities of intra-acrosomal enzymes in spermatozoa before and after freezing and found that acrosin-binding protein and triosephosphate isomerase showed significant differences in their activities before and after freezing and that these two enzymes may be related to spermatozoa activity. Pinart et al (Pinart et al., 2013). found that elevated temperature and radiation significantly affected acrosin activity. Pregnancy rate and litter size were significantly correlated with acrosin activity. Based on this, sperm motility and its membrane integrity were investigated, and it was found that acrosin activity had a significant difference between good and poor freezing performance (Pinart et al., 2015). These studies suggest that acrosin activity is a good indicator of frozen sperm quality evaluation. Acrosin is now applied to determine the quality of frozen sperm in boars (Pinart et al., 2015), Russian sturgeons (Huang et al., 2021), dogs (De Los Reyes et al., 2011), arctic foxes (Stasiak et al., 2014), raccoon dogs (Jarosz et al., 2016), and stallions (Sales et al., 2018).

### 8.2 Testing of sperm quality in males

Bartoov et al (Bartoov et al., 1994). found a correlation between human sperm acrosin activity and sperm *in vitro* fertilization ability, with a significant positive correlation between acrosin levels and fertility status. Acrosin activity may be a clinical laboratory indicator for assessing the fertilization potential of spermatozoa (Agarwal and Loughlin, 1991). found that 10 out of 15 infertility patients had acrosin levels in the low fertility range, whereas all four fertile men had acrosin levels in the normal range (Cui et al., 2000). compared acrosin activity in normal fertile and infertile males and showed that acrosin activity was higher in normal fertile men than in infertile men, and there was a significant positive correlation between acrosin activity and sperm motility. These results indicate that the content and activity of acrosin are related to male sperm motility. It has also been found that sperm motility was high in patients with unexplained infertility, which could affect the proacrosin-acrosin system. Therefore, the total acrosin activity could be used as an indicator for the clinical evaluation of unexplained causes of infertility (Chaudhury et al., 2005). (Xu et al., 2018a) conducted a retrospective study on the correlation between the levels of acrosomal protease (including acrosin and hyaluronidase), measured using spectrophotometry and fertilization rate; this correlation was significant. The results indicate that acrosin levels could be used to predict sperm quality; however, this detection method was not highly sensitive or specific.

### 8.3 Application of acrosin to the development of new contraceptives

Acrosin can stimulate sperm to penetrate the zona pellucida to complete fertilization, and the absence of acrosin causes incomplete fertilization. Therefore, acrosin is an ideal target for contraceptive medicine. Researchers have developed novel acrosin inhibitors such as 7-azaindole derivatives, 5-phenyl-1H-pyrazole-3-carboxylic acid amide derivatives, 5-(4-aminophenyl)-1H-pyrazole-3-carboxylate derivatives, novel quinazolinone derivatives, and novel guanidinophenyl pyrazole, which are the basis for developing new contraceptives (Jiang et al., 2011; Ning et al., 2013; Qi et al., 2011; Tian et al., 2013; Zhao et al., 2014).

## 9 Conclusion

Since the first discovery of acrosin as an influential factor in the promotion of sperm fertilization, its isolation, purification, structure, and properties have been studied for more than 80 years. During this time, researchers have conducted extensive studies on the isolation and purification of various animal acrosin, which have been isolated and purified primarily using acid extraction and a combination of multiple chromatographic methods to obtain animal sperm acrosomal enzymes of high purity. Therefore, further studies on their structural and enzymatic properties have contributed to a greater understanding of this type of enzyme. Based on the complete understanding of the structure and properties of acrosin, these enzymes have been rationally applied to different fields, such as sperm cryopreservation, mammalian fertility evaluation, and the development of novel contraceptives.

Acrosin is involved in the fertilization of animal sperm cells and oocytes, can facilitate sperm penetration of the oocyte zona pellucida, and is a key enzyme in the fertilization process. However, it has been discovered recently that acrosin defects do not cause sterility in male mice but cause unfertilized oocytes in humans. Thus, the mechanism of acrosin action in different species to promote spermatozoa and complete fertilization needs to be further investigated.
